# Skin bioprinting: the future of burn wound reconstruction?

**DOI:** 10.1186/s41038-019-0142-7

**Published:** 2019-02-12

**Authors:** Mathew Varkey, Dafydd O. Visscher, Paul P. M. van Zuijlen, Anthony Atala, James J. Yoo

**Affiliations:** 1 Wake Forest Institute for Regenerative Medicine, Wake Forest School of Medicine, Medical Center Boulevard, Winston-Salem, NC 27101 USA; 2 Department of Plastic, Reconstructive and Hand Surgery, Amsterdam University Medical Center, 1081 HV Amsterdam, The Netherlands; 3 Amsterdam Movement Sciences, Amsterdam, The Netherlands; 4 Burn Center, Red Cross Hospital, 1942 LE Beverwijk, The Netherlands; 5 Association of Dutch Burn Centres, 1942 LE Beverwijk, The Netherlands

**Keywords:** Bioprinting, Skin, Burns, Reconstruction

## Abstract

Burns are a significant cause of trauma, and over the years, the focus of patient care has shifted from just survival to facilitation of improved functional outcomes. Typically, burn treatment, especially in the case of extensive burn injuries, involves surgical excision of injured skin and reconstruction of the burn injury with the aid of skin substitutes. Conventional skin substitutes do not contain all skin cell types and do not facilitate recapitulation of native skin physiology. Three-dimensional (3D) bioprinting for reconstruction of burn injuries involves layer-by-layer deposition of cells along with scaffolding materials over the injured areas. Skin bioprinting can be done either in situ or in vitro. Both these approaches are similar except for the site of printing and tissue maturation. There are technological and regulatory challenges that need to be overcome for clinical translation of bioprinted skin for burn reconstruction. However, the use of bioprinting for skin reconstruction following burns is promising; bioprinting will enable accurate placement of cell types and precise and reproducible fabrication of constructs to replace the injured or damaged sites. Overall, 3D bioprinting is a very transformative technology, and its use for wound reconstruction will lead to a paradigm shift in patient outcomes. In this review, we aim to introduce bioprinting, the different stages involved, in vitro and in vivo skin bioprinting, and the various clinical and regulatory challenges in adoption of this technology.

## Background

Burns are amongst the most common types of trauma worldwide. More than 11 million people require burn-related medical attention each year [1]. Most burn injuries occur in a domestic setting in low- and middle-income countries, but industrial accidents and armed conflicts also contribute to the high incidence of burns [2]. Since the twentieth century, the number of serious burns has decreased dramatically due to increased prevention [3]. Advances in burn injury knowledge, multidisciplinary and better burn critical care, and pharmacological developments in the last few decades have resulted in a shift in attention from mortality to the functional recovery following burns [4, 5]. The focus of patient care has shifted from merely survival to accelerated wound closure, improved scar quality, and functional outcomes [4].

## Review

### Reconstructive surgery for burn treatment

There are several surgical procedures to treat burn wounds including primary closure, burn wound excision with subsequent skin grafts, and skin substitutes. Currently, most burn patients survive their injuries. Following the initial resuscitation and stabilization of the burn patient [6, 7], surgical wound closure and reconstructive surgery are typically performed to improve the functional and esthetic outcomes of burn wounds.

Primary closure of burn wounds involves direct wound closure following excision of the devitalized tissue. It is usually performed in small- to moderate-sized burn scars and takes into account Langer’s lines of skin tension for an optimal esthetic outcome [8]. Recently, primary closure has also been performed in larger burn wounds in combination with skin-stretching devices [9–12].

When primary closure of a burn wound is not an option, additional surgery is required. A combination of excision and grafting is the preferred approach for the treatment of deeper dermal burns. The main goal of early excision is to remove devitalized tissue and prepare the wound for skin grafting; layers of burned tissue are excised until a viable wound bed is reached for grafting [3]. Early excision has been shown to be cost effective and reduce mortality and the length of hospital stay [13, 14].

Covering the excised burn wound with autologous skin grafts harvested from an uninjured donor site on the patient is considered as the gold standard for repair of burn injuries. These autologous skin grafts can either be partial (split-thickness skin grafts (STSGs)) or full-thickness skin grafts (FTSGs), depending on the thickness of the obtained graft. STSGs consist mostly of the epidermis, while FTSGs consist of both epidermis and dermis. Although STSGs are the gold standard for autografts in burn surgery because of their versatility and self-regenerating capacity, FTSGs are often preferred over STSGs since they tend to give better esthetic results with less contraction [15]. However, a recent study reported that FTSGs also show significant long-term surface area reduction [16]. For smaller injuries, hand and facial burns, and burns in children, STSGs are preferred [6]. Functional outcome is often related to the availability of donor skin for reconstruction and the prevention of extensive scarring and skin contracture. The preferred initial treatment of deeper dermal burns includes early excision and grafting. Despite the advantages and disadvantages of both STSGs and FTSGs, donor skin is often limited in patients with severe burns [17, 18]. Although repeated harvesting of a donor site over time can be an option, it can cause scarring and pigmentation disorders [19, 20]. Another option is to increase the surface area of skin grafts by graft expansion. Graft expansions can be prepared using mesh techniques [21–23] or the (modified) Meek technique [24–27]. However, in the case of extensive skin loss such as cases where skin loss exceeds 60% of the total body surface area of the patient, the availability of donor sites for harvesting is severely limited [28–30]. In addition, autografting generates donor sites which are not only painful during healing but may also develop scar and cause long-term morbidity. Other types of skin grafts such as allogeneic skin transplants from non-genetically identical individuals or cadaver skin and xenogeneic skin transplants from different species serve only as temporary treatment measures for full-thickness wounds [31]. They require resurfacing with an autogenous epidermal layer because of immunologic rejection or rejection due to host immune response elicited by antigens present in the donor tissue. Tissue-engineered skin substitutes are a promising alternative. They typically consist of allogeneic cells that provide temporary protection to the wounds or autologous epidermal keratinocytes and dermal fibroblasts applied as cell sprays or as cultured tissue constructs to facilitate wound closure and healing. Skin substitutes such as Integra®, Biobrane®, Dermagraft®, and Apligraf® are already employed in the clinic, with or without complementation of autologous STSGs[31]. These substitutes have been shown to effectively close full-thickness burn wounds and enable survival after life-threatening burn injuries.

There are currently a wide range of different skin substitutes available for clinical applications [19, 32], the majority of which are biosynthetic skin substitutes (e.g., Matriderm®, Integra®, Dermagraft®, and OrCel®) [33–37]. Even though the use of skin substitutes is still investigational, many burn clinics use skin substitutes for the treatment of burns. For example, the application of Integra®, a biosynthetic dermal scaffold consisting of bovine type I collagen and chondroitin-6-sulfate, may result in improved scar appearance and elasticity and less donor site morbidity [38–40]. In addition, Matriderm®, an intact matrix of bovine type I collagen and elastin, was shown effective in pilot trials and resorbs as the wound healing process advances [33]. Despite good clinical results, there are still many challenges regarding skin substitutes. For example, the majority of skin substitutes consist of allogeneic skin which can be highly immunogenic and contain cellular remnants that may cause rejection of the skin substitute [41]. In addition, methods to sterilize skin substitutes may be insufficient to eliminate the transmission of unknown or prion disease(s) from animal material [42]. Furthermore, human-derived skin is limited by its supply, and the structure is a lot more complex than biosynthetic substitutes. Finally, although most skin substitutes perform relatively well in the clinic, these substitutes do not include hair and pigment, which are both important for the normal functions of the skin [43].

### Disruptive technology in burn care

Conventional tissue-engineered skin substitutes are made by seeding cells on biodegradable scaffolds and allowed to mature, following which they are used for transplantation or in vitro testing. These skin substitutes have several limitations, they contain at most only two cell types, and since they are based on post-natal wound healing physiology, they do not stimulate regeneration of vasculature, nerves, sweat and sebaceous glands, hair follicles, and pigmentation. All these structures are essential to restore the complete anatomy and physiology of native skin; hence, there is an immense need to develop next generation tissue-engineered skin substitutes. Recent work from our group demonstrates that bioprinting could be successfully used to close large full-thickness wounds [44]. Further, we have also shown that bioprinting could be very effectively used to precisely fabricate both soft and hard tissues with complex structures in an automated manner [45]. Bioprinting could revolutionize the field of burn care by replacing current off-the-shelf cellular or acellular skin products and providing highly automated process of fabricating complex skin constructs to enhance functional outcome of burns. In this review, we discuss current developments in skin bioprinting for burn reconstruction and highlight the challenges that need to be addressed in the coming years.

### Three-dimensional (3D) bioprinting

**Fig. 1:**
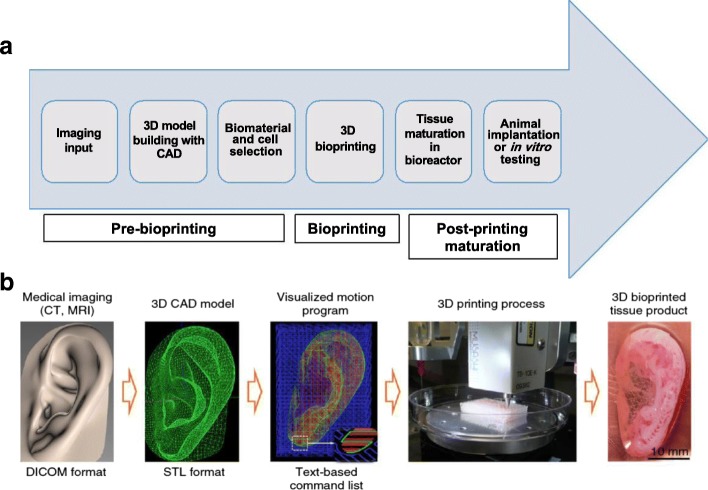
The bioprinting process. **a** Different steps and stages that lead to the production of bioprinted constructs for implantation or in vitro testing. **b** The process of bioprinting using the integrated tissue and organ printer illustrated using ear cartilage bioprinting. This figure was adapted from the original article of Kang et al. [45] (Copyright 2016 Nature America, Inc.). Data from the medical imaging input is used to generate the 3D CAD model. A visualized motion program is generated, and instructions to print the construct are transmitted to the computer using a text-based command. *3D* three-dimensional, *CAD* computer-aided design, *MRI* magnetic resonance imaging, *CT* computed tomography, *STL* STereoLithography, *DICOM* digital imaging and communications in medicine

3D printing involves sequential delivery of thin layers of materials and bonding them together to form a solid 3D structure [46]. First developed by Charles W. Hull in 1986 and originally called “stereolithography”, 3D printing is an additive manufacturing technique [46]. 3D printing can automate tissue engineering and facilitate cost-effective large-scale manufacturing. 3D bioprinting, a variant of 3D printing, is a computer-aided manufacturing process that deposits living cells together with hydrogel-based scaffolds (also called “bioink”) and allows for patterning of individual components of the tissue or organ, thereby facilitating formation of complex tissue architecture [47]. Fabrication of biological constructs by 3D bioprinting typically involves layer-by-layer addition of material on a supporting scaffold to build 3D tissue with input from a computer-aided design (CAD) file [48]. Bioprinting enables tailor fabrication of tissue constructs by suitably altering the CAD file prior to printing [49]. Generally, the process of 3D bioprinting involves five different steps: (1) imaging/scanning of the target tissue is performed; (2) using the imaging input, the model is developed with CAD-CAM (computer-aided manufacture) softwares; (3) depending on the tissue to be printed, the biomaterial scaffolds and cells are carefully chosen, one or more cell types could be used; (4) the tissue is printed using a bioprinter; and (5) the bioprinted tissue is allowed to mature. Bioprinting can be done in vitro or in situ; if it is done in vitro, following tissue maturation, the bioprinted tissue constructs are used either for implantation or in vitro testing [46, 50] (Fig. 1a and b). Broadly, the bioprinting process proceeds in three different stages: the tissue pre-bioprinting, bioprinting, and post-bioprinting maturation stages.

**Fig. 2:**
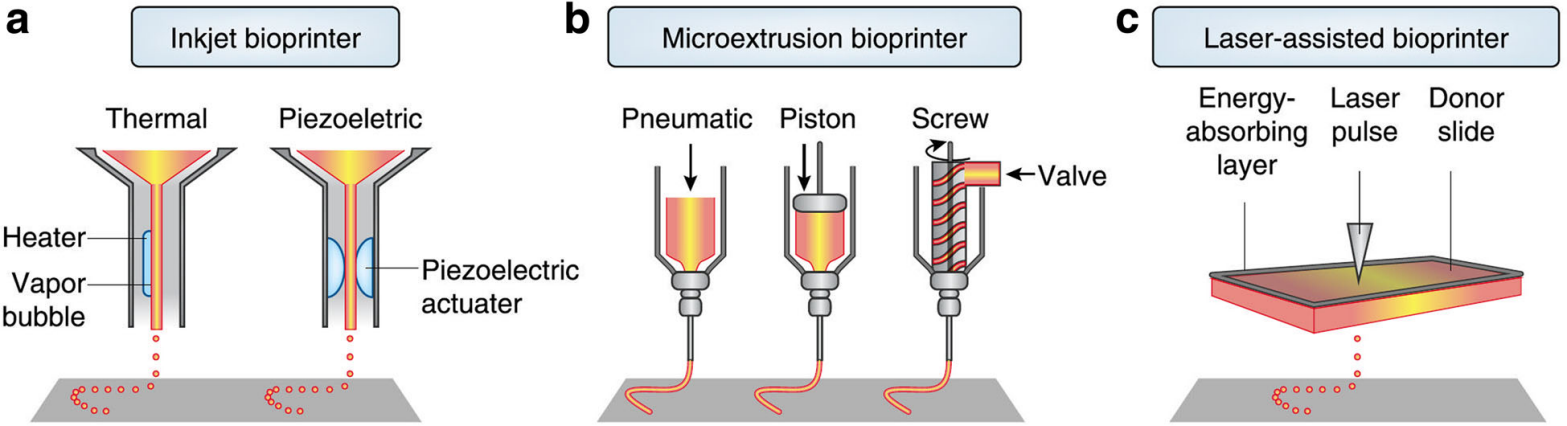
Components of inkjet, microextrusion, and laser-assisted bioprinters. This figure was adapted from the original article of Murphy et al. [46] (Copyright 2014 Nature America, Inc.). **a** In thermal inkjet printers, the print head is electrically heated to produce air-pressure pulses that force droplets from the nozzle, while acoustic printers use pulses formed by piezoelectric or ultrasound pressure. **b** Microextrusion printers use pneumatic or mechanical dispensing systems to extrude continuous beads of material and/or cells. **c** Laser-assisted printers use lasers focused on an absorbing substrate to generate pressures that propel cell-containing materials onto a collector substrate

The main technological systems for bioprinting include inkjet-, microextrusion- and laser-based bioprinting [46] (Fig. 2). Inkjet-based bioprinting utilizes thermal-, piezo-, or acoustic-driven mechanisms to deposit droplets of cell suspension in a high-throughput manner [46]. While there are many advantages to the inkjet bioprinting technology, a downside is the risk of exposing cells and materials to thermal and mechanical stress, and in the case of acoustic printers, the use of high frequencies may affect cell viability. Inkjet bioprinters are also limited by the viscosity of the bioink used; the more viscous the bioink the greater the force required to eject the droplet from the printer nozzle [46]. Further, the cell density that can be used for printing may be lower than physiologically relevant numbers due to the possible nozzle clogging issues.

Microextrusion bioprinting uses mechanical or pneumatic dispensing systems to extrude continuous beads of materials that consist of cells mixed with hydrogels [46]. Structures are printed with hydrogel, and the material is then solidified either physically or chemically such that the structures can be combined to create 3D shapes. Microextrusion printers allow for a wider selection of biomaterials since more viscous materials can be printed. Another advantage is that these printers can deposit very high cell densities. Although cell viability may be lower than that obtained with inkjet printers, it is in the range of 40 to 86%, depending on the size of nozzle and pressure of extrusion used [51].

Laser-assisted bioprinting is another type of printing system which is based on the principles of laser-induced forward transfer. This involves the use of a pulsed laser beam, a focusing system and a “ribbon” that has a donor transport support, a layer of biological material, and a receiving substrate facing the ribbon [48, 52]. Focused laser pulses are used to generate a high-pressure bubble that propels cell-containing materials toward the collector substrate. Since laser bioprinting does not use nozzles, there are no cell clogging issues. Another advantage is the ability to print with high cell densities without affecting cell viability [53, 54]. The main disadvantages however are the reduced overall flow rate as a result of the high resolution and also the possibility of metallic residues in the final construct [48, 55].

In addition to laser-assisted bioprinting, other light-based 3D bioprinting techniques include digital light processing (DLP) and two-photon polymerization (TPP)-based 3D bioprinting. DLP uses a digital micro-mirror device to project a patterned mask of ultraviolet (UV)/visible range light onto a polymer solution, which in turn results in photopolymerization of the polymer in contact [56, 57]. DLP can achieve high resolution with rapid printing speed regardless of the layer’s complexity and area. In this method of 3D bioprinting, the dynamics of the polymerization can be regulated by modulating the power of the light source, the printing rate, and the type and concentrations of the photoinitiators used. TPP, on the other hand, utilizes a focused near-infrared femtosecond laser of wavelength 800 nm to induce polymerization of the monomer solution [56]. TPP can provide a very high resolution beyond the light diffraction limit since two-photon absorption only happens in the center region of the laser focal spot where the energy is above the threshold to trigger two-photon absorption [56].

The recent development of the integrated tissue and organ printer (ITOP) by our group allows for bioprinting of human scale tissues of any shape [45]. The ITOP facilitates bioprinting with very high precision; it has a resolution of 50 μm for cells and 2 μm for scaffolding materials. This enables recapitulation of heterocellular tissue biology and allows for fabrication of functional tissues. The ITOP is configured to deliver the bioink within a stronger water-soluble gel, Pluronic F-127, that helps the printed cells to maintain their shape during the printing process. Thereafter, the Pluronic F-127 scaffolding is simply washed away from the bioprinted tissue. To ensure adequate oxygen diffusion into the bioprinted tissue, microchannels are created with the biodegradable polymer, polycaprolactone (PCL). Stable human-scale ear cartilage, bone, and skeletal muscle structures were printed with the ITOP, which when implanted in animal models, matured into functional tissue and developed a network of blood vessels and nerves [45]. In addition to the use of materials such as Pluronic F-127 and PCL for support scaffolds, other strategies for improving structural integrity of the 3D bioprinted constructs include the use of suitable thickening agents such as hydroxyapatite particles, nanocellulose, and Xanthan and gellan gum. Further, the use of hydrogel mixtures instead of a single hydrogel is a helpful strategy. For example, the use of gelatin-methacrylamide (GelMA)/hyaluronic acid (HA) mixture instead of GelMA alone shows enhanced printability since HA improves the viscosity of mixture while crosslinking of GelMA retains post-printing structural integrity [58].

### Skin bioprinting—in situ and in vitro

To date, several studies have investigated skin bioprinting as a novel approach to reconstruct functional skin tissue [44, 59–67]. Some of the advantages of fabrication of skin constructs using bioprinting compared to other conventional tissue engineering strategies are the automation and standardization for clinical application and precision in deposition of cells. Although conventional tissue engineering strategies (i.e., culturing cells on a scaffold and maturation in a bioreactor) might currently achieve similar results to bioprinting, there are still many aspects that require improvements in the production process of the skin, including the long production times to obtain large surfaces required to cover the entire burn wounds [67]. There are two different approaches to skin bioprinting: (1) in situ bioprinting and (2) in vitro bioprinting. Both these approaches are similar except for the site of printing and tissue maturation. In situ bioprinting involves direct printing of pre-cultured cells onto the site of injury for wound closure allowing for skin maturation at the wound site. The use of in situ bioprinting for burn wound reconstruction provides several advantages, including precise deposition of cells on the wound, elimination of the need for expensive and time-consuming in vitro differentiation, and the need for multiple surgeries [68]. In the case of in vitro bioprinting, printing is done in vitro and the bioprinted skin is allowed to mature in a bioreactor, after which it is transplanted to the wound site. Our group is working on developing approaches for in situ bioprinting [69]. An inkjet-based bioprinting system was developed to print primary human keratinocytes and fibroblasts on dorsal full-thickness (3 cm × 2.5 cm) wounds in athymic nude mice. First, fibroblasts (1.0 × 10^5^ cells/cm^2^) incorporated into fibrinogen/collagen hydrogels were printed on the wounds, followed by a layer of keratinocytes (1.0 × 10^7^ cells/cm^2^) above the fibroblast layer [69]. Complete re-epithelialization was achieved in these relatively large wounds after 8 weeks. This bioprinting system involves the use of a novel cartridge-based delivery system for deposition of cells at the site of injury. A laser scanner scans the wound and creates a map of the missing skin, and fibroblasts and keratinocytes are printed directly on to this area. These cells then form the dermis and epidermis, respectively. This was further validated in a pig wound model, wherein larger wounds (10 cm × 10 cm) were treated by printing a layer of fibroblasts followed by keratinocytes (10 million cells each) [69]. Wound healing and complete re-epithelialization were observed by 8 weeks. This pivotal work shows the potential of using in situ bioprinting approaches for wound healing and skin regeneration. Clinical studies are currently in progress with this in situ bioprinting system. In another study, amniotic fluid-derived stem cells (AFSCs) were bioprinted directly onto full-thickness dorsal skin wounds (2 cm × 2 cm) of nu/nu mice using a pressure-driven, computer-controlled bioprinting device [44]. AFSCs and bone marrow-derived mesenchymal stem cells were suspended in fibrin-collagen gel, mixed with thrombin solution (a crosslinking agent), and then printed onto the wound site. Two layers of fibrin-collagen gel and thrombin were printed on the wounds. Bioprinting enabled effective wound closure and re-epithelialization likely through a growth factor-mediated mechanism by the stem cells. These studies indicate the potential of using in situ bioprinting for treatment of large wounds and burns.

There are a few reports of in vitro skin printing from other groups. Laser-assisted bioprinting was used to print fibroblasts and keratinocytes embedded in collagen and fabricate simple skin equivalent structures [64]. The cells were shown to adhere together through the formation of gap junctions. In a similar study, fibroblasts and keratinocytes were printed in vitro on Matriderm® stabilizing matrix [63]. These skin constructs were subsequently tested in vivo, using a dorsal skin fold chamber model in nude mice. On full-thickness wounds, a multilayer epidermis with stratum corneum was observed in the explanted tissue after 11 days. Also, at this time, some blood vessels were found to be arising from the wound bed. In another report, dermal/epidermal-like distinctive layers were printed using an extrusion printer with primary adult human dermal fibroblasts and epidermal keratinocytes in a 3D collagen hydrogel. Epidermal and dermal structures were observed in these constructs; however, they did not show establishment of intercellular junctions [70]. More recently, Cubo et al. printed a human plasma-derived skin construct with fibroblasts and keratinocytes [67]. The printed skin was analyzed in vitro and in vivo in an immunodeficient mouse model. The printed skin had a structure similar to native skin with identifiable stratum basale, stratum granulosum, and stratum corneum suggesting a functional epidermal layer and neovascular network formation [67]. In order to regenerate fully functional skin using bioprinting, other structures such as skin appendages (e.g., hair follicles, sweat glands, melanocytes, endothelial cells, and sebaceous glands) should be co-printed in the skin. Some recent studies have evaluated printing of melanocytes [62] and sweat glands [71, 72] with varying results. Min and colleagues [62] co-printed melanocytes and keratinocytes on top of a dermal layer and showed terminal differentiation of keratinocytes and freckle-like pigmentations without the use of UV light or chemical stimuli. Huang and colleagues [72] bioprinted sweat glands using epidermal progenitor cells in a composite hydrogel based on gelatin and sodium alginate. They showed that the bioprinted 3D extracellular matrix (ECM) resulted in functional restoration of sweat glands in burned mice.

#### Stages of skin bioprinting

The process of skin bioprinting can be divided into three stages: (1) skin pre-printing, (2) bioprinting, and (3) skin maturation. Pre-printing involves isolation of cells from the skin biopsy, expansion of cells, differentiation of cells, and preparation of the bioink, which is made of cells and biomaterial support materials. In the case of healthy skin, primary cells could be isolated, expanded, and used; however, in the case of injured skin, stem cells may need to be differentiated into epidermal and mesenchymal cells. Stem cells can be obtained from different sources including adipose, mesenchymal, perinatal, and induced pluripotent stem cells. For bioprinting, the print files that contain accurate surface information of complex 3D geometries are converted to the STereoLithography (STL) file format with coordinates for the print head path [47, 73]. These files contain accurate surface information required to reconstruct the complex 3D model and can be designed using CAD-CAM graphic user interfaces or created from clinical images with input from magnetic resonance imaging (MRI) and computed tomography (CT) imaging [74, 75]. The paths for the print heads are created by slicing the STL model into layers and creating bioprinter toolpaths that trace out the perimeter and interior features of each slice. The thickness of each of these slices determines the resolution of the printer and is usually in the 100–500 μm range. Resolution is specific to the printer used; the smaller the resolution the better the quality but longer the print time. The bioprinter reads the STL files and layer-by-layer deposits the bioink to build the 3D tissue or organ from the series of 2D slices. High-quality image acquisition is essential for high-fidelity bioprinting. Clinical images can provide information regarding the in vivo cell distribution, and image processing tools can be used to determine anatomically realistic skin geometry. The final stage of bioprinting is the maturation stage. This is especially critical in case of in vitro bioprinting, and immediately following printing, the skin constructs are fragile and need to be matured in a bioreactor for a few days prior to use for transplantation. When the skin is in situ bioprinted, maturation occurs on the body at the site of injury.

#### Bioink—the essential element for bioprinting

Bioinks form the delivery medium that encapsulates the cells, minimize cell injury during the printing process, and provide a supportive microenvironment for maturation of the bioprinted skin. The choice of bioink is a critical aspect of bioprinting essential for the different cells to be deposited in specific patterns of the CAD models and is chosen with the desired biomechanical characteristics in mind. An appropriate choice of bioink is essential to provide the chemical and physical cues that facilitate necessary cell-ECM interactions; bioink properties not only affect cell growth, proliferation, and differentiation but also structure and function of the bioprinted skin. It is essential that the chosen bioink be biocompatible and cell supportive and facilitate functional differentiation of the cells into the skin [76]. Typically, the bioinks could physically serve as cell-laden hydrogels or sacrificial support materials that are removed immediately after printing or as mechanical support materials that provide specific mechanical characteristics to the tissue. Bioinks can be fully natural materials such as collagen, fibrin, HA, and alginate, which could be used in the form of hydrogels for the cells or synthetic materials such as PCL, polylactide (PLA), polyglycolide (PGA), poly(lactic-co-glycolic acid) (PLGA), and polyethylene glycol (PEG) polymers or hybrid biomaterials that contain a combination of natural and synthetic materials, which could provide mechanical support [77]. Other bioinks that are typically used also include agarose-, silk-, cellulose-, and GelMA)-based bioinks. Materials such as Pluronic F-127 could be used as sacrificial support materials that keep the cells together while printing and could be simply washed away following printing of the tissue construct [45].

#### Features of bioink

Printability of the bioink indicates the ease with which it could be printed with good resolution and its ability to maintain its structure for post-printing skin maturation. The bioink formulation should be stable enough to provide architectural stability to the skin construct. Shape fidelity and printing resolution are important considerations when assessing the printability of the bioink [78]. Other important bioink properties to consider include gelation kinetics, rheological characteristics, and material properties. Ideally, the viscosity of the bioink should be such that it is not only supportive for cell growth and differentiation but also suitable for printing, but in reality viscosities appropriate for bioprinting may not be supportive of cell viability. So, to achieve good printability and at the same time to ensure high cell viability, the printing conditions and bioink consistency need to be optimized. The biomechanical and structural characteristics of the skin are also important considerations for choice of bioink. As we advance in our ability to bioprint and potentially attempt to bioprint composite tissue that may contain a mix of soft and hard tissue such as the skin, skeletal muscle, and bone, we will need to develop some sort of standard or universal bioink that could support different tissue types without compromising functionality. Another important factor that should be considered is how quickly the material will degrade in the body; the cells should be able to degrade the scaffold at a rate that will match their ECM production and remodeling activity. For recent advances in the area of bioinks, we refer readers to recent reviews about the subject [79, 80].

### Considerations for bioprinting skin

**Fig. 3:**
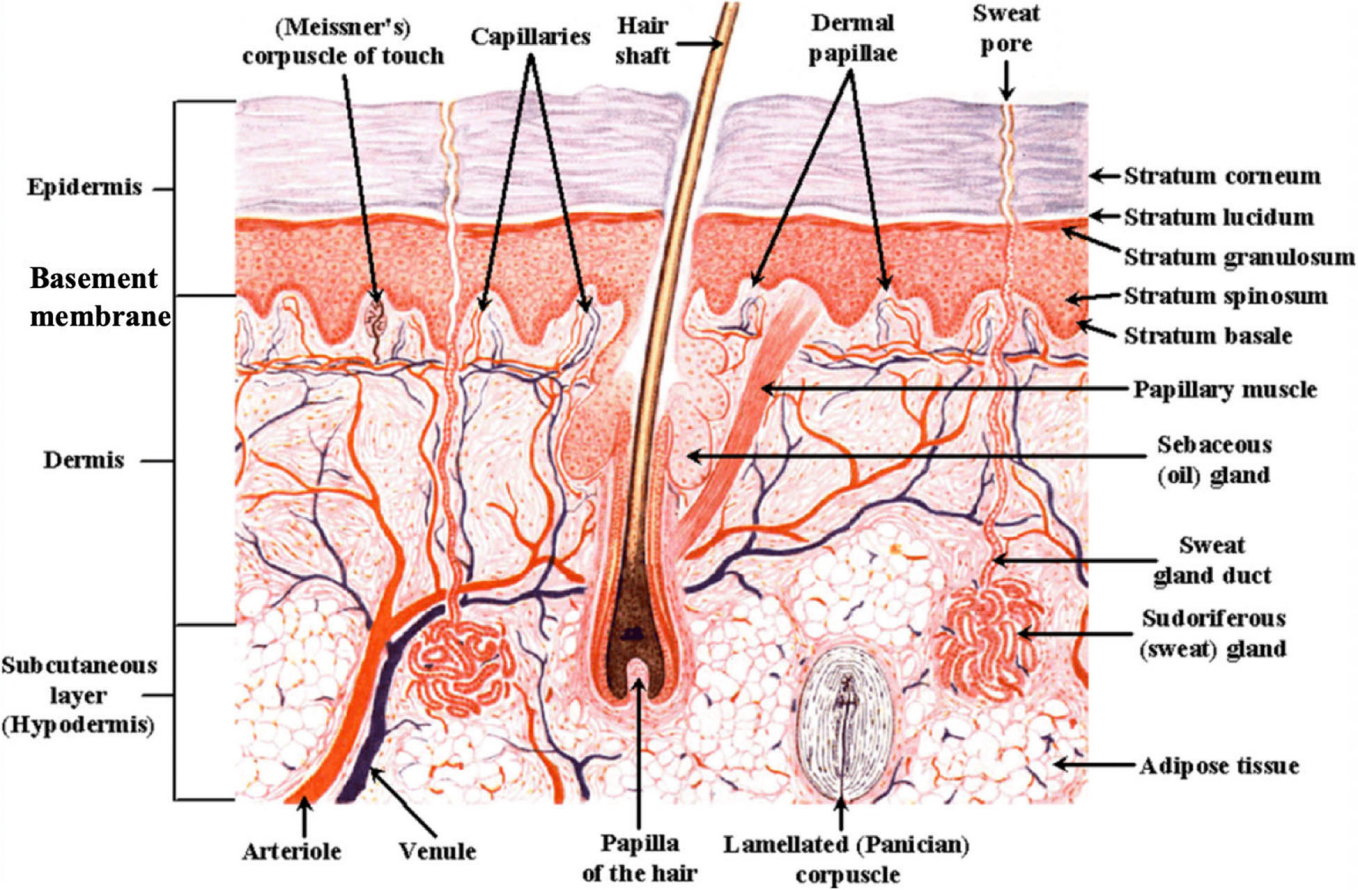
Structure of human skin depicting the different layers and appendages

The skin is a complex organ with a well-defined structure consisting of multiple layers and appendages and is made of several cell types (Fig. 3). Therefore, to bioprint such a structure requires multiple cell types and biomaterials. The most superficial layer of the skin, the epidermis, is mainly composed of keratinocytes with varying degrees of differentiation and intertwined melanocytes near the lower layer of the epidermis. The epidermis is relatively thin (0.1–0.2 mm in depth) and attached to the underlying dermis via a highly specialized basement membrane [81]. Due to the relatively thin epidermis, laser-assisted bioprinting technology may be used to explore epidermal bioprinting [82]. Utilizing this technology, one may be able to recapitulate the epidermal morphology by printing consecutive layers of keratinocytes and melanocytes. The bioprinting technology could potentially be used to produce uniform pigmentation in patients [83]. The basement membrane is a thin, fibrous tissue composed of two layers, the basal lamina and the reticular connective tissue, which are connected with collagen type VII anchoring fibrils and fibrillin microfibrils [84]. The structure of the basement membrane becomes more complex deeper in the skin, where the tissue becomes several nanometers thick with many ECM components including collagen type IV, laminin, and various integrins and proteoglycans [84]. Bioprinting such a complex layer is a challenging and complex task, and therefore many researchers tend to rely on tissue self-assembly after printing [85, 86].

The dermal layer can be found directly underneath the basement membrane in the skin and is composed of fibroblasts embedded in a complex ECM [28]. This layer also contains many different structures including all skin appendages, blood vessels, and nerves, which serve the epidermis. The reticular or deep dermis contains many ECM components including collagen and elastin; these elastic and reticular fibers give the skin its high elasticity and strength. In addition, the organization of these fibers also creates Langer’s lines [8]. Therefore, this structure may be very important for the mechanical stability of bioprinted skin. Because this layer is thicker than the overlying epidermis, extrusion-based technology may be a good option as it can combine multiple cell types and biomaterials. The use of bioprinting will enable incorporation of other cell types in the dermis including hair follicles and sweat and sebaceous glands. This will enable regeneration of the skin tissue with structure and cellular composition resembling native tissue. In addition, bioprinting will enable control of the microarchitecture of the dermal tissue components, which may have a role in the formation of scar during the wound repair and healing process following injury [87].

Tailoring the microenvironment to facilitate tissue regeneration over repair may have some benefits in terms of better functional outcomes during the scar remodeling process [87]. The hypodermis lies directly below the dermis and consists mainly of adipose tissue that provides heat insulation, energy storage, protective padding [88], and a sliding system [89, 90]. This last function has only recently become important in burn surgery because restoring the burned hypodermis with autologous fat injection has shown a remarkable improvement in scar pliability [90].

### Technological challenges

To enable clinical translation of bioprinting technology, several technological limitations at the pre-printing, bioprinting, and maturation stages of the bioprinting process need to be overcome [91].

Very large numbers of cells are required for printing transplant-ready skin; to bioprint skin with physiologically-equivalent cell numbers, billions of cells will be needed. Current cell expansion technologies facilitate cell expansion in the range of millions, so innovative cell expansion technologies need to be developed [79]. Further, development of superior bioinks that allow for reproducible bioprinting of the skin with appropriate biomechanical properties is critical for clinical translation of the technology.

For composite tissue that contains different tissue types, the printing resolution will need to be improved to duplicate the intricate inner microarchitecture. The ability to print microscale features is necessary for optimal cellular function. Better control over the microarchitecture will enable fabrication of the skin capable of recapitulating the native form and function. Increasing the printing speed is another challenge; current approaches that facilitate higher printing speed such as extrusion bioprinting can compromise the integrity of cells and cause significant loss in their viability. CAD-CAM can also be used to predict the feasibility of the fabrication process by simulating relevant physical models using both classical formula calculations and finite element methods. Currently, the most widely used physical model for bioprinting is laminar multi-phase flow; although it is an oversimplified model and ignores issues related to inclusion of cells, the simulations are useful for checking and optimizing the feasibility of specific designs.

Building a functional vasculature is one of the most fundamental challenges in tissue engineering. The ability to 3D bioprint vasculature will enable fabrication of a preformed microvascular network that can better anastomose to the host circulation and achieve functional perfusion within the tissue-engineered skin construct [92, 93]. The use of sacrificial inks to create 3D interconnecting networks, which can be removed after printing the entire construct, leaving hollow channels for the perfusion of endothelial cells and formation of blood vessel network is a promising approach. Miller et al. have shown how 3D extrusion printing and cast molding could be combined to create a 3D-interconnected perfusable vasculature [94]. However, this molding technique is limited to the construction of simple block tissue architectures [94]. Recently, a bioprinting approach that enables the simultaneous printing of the vasculature structure and the surrounding cells for heterogeneous cell-laden tissue constructs has been reported by the research group of Prof. Lewis [95]. They have developed a method that involves the use of Pluronic F-127 as a fugitive bioink, which can be printed and dissolved under mild conditions, enabling printing of heterogeneous cell-laden tissue constructs with interconnecting vasculature networks [95].

There have also been attempts to bioprint the vascular network directly; Zhang et al. recently reported about direct bioprinting of vessel-like cellular microfluidic channels with hydrogels, such as alginate and chitosan, using a coaxial nozzle [96]. In very recently reported work from Prof. Lewis’ lab, they have demonstrated bioprinting of 3D cell-laden, vascularized tissues that exceed 1 cm in thickness and can be perfused on chip for greater than 6 weeks [97]. They integrated parenchyma, stroma, and endothelium into a single thick tissue by co-printing multiple inks composed of human mesenchymal stem cells and human neonatal dermal fibroblasts within a customized fibrin-gelatin matrix alongside embedded vasculature, which was subsequently lined with human umbilical vein endothelial cells. This may open newer avenues for printing of pre-vascularized skin tissue.

To print vascularized skin models with complexity and resolution matching in vivo structures, print resolution needs to be improved and printing time reduced. The ability to bioprint hierarchical vascular networks while building complex tissues and the ability to recapitulate vascular flow in vitro [98] are critical for fabrication of transplantable organs.

Native skin has different cell types, each of them require different nutritional and metabolic support. Development of a standard or universal growth media for cells will be beneficial for growth and maturation of composite tissue constructs prior to transplantation. The cells also are in dynamic reciprocity with their microenvironment, which includes the ECM in which they are embedded in. The cells secrete proteins, proteases, and other metabolites onto the ECM, which facilitate dynamic homeostatic phase of tissue remodeling. Inclusion of native ECM in the bioink will ensure the presence of natural ligands and thus facilitate a suitable growth environment for the cells [79]. Also, the development of novel bioreactors to facilitate dynamic culture would facilitate physiologic-like environment for the maturation of tissues that incorporate printed vasculatures [79].

In the future, better analytical and computational approaches to effectively study the development and maturation of the bioprinted tissue prior to transplantation need to be developed [79]. There has been a lot of effort to model bioprinted tissue with the corresponding printing parameters. For extrusion printing, relationships between dispensing pressure, printing time, and nozzle diameter have been tested and modeled [89]. In inkjet printers, cell settling that occurs during printing and is known to cause clogging of the nozzles has been modeled by both analytical and finite element methods [92–94]. For laser printing, the effects of laser energy, substrate film thickness, and hydrogel viscosity on cell viability [95] as well as droplet size [54, 94], cell differentiation [96], and cell proliferation [96] have been studied. Researchers have also done post-printing modeling of cellular dynamics [97, 98], fusion [98], deformation, and stiffness [99].

### Clinical and regulatory requirements

Efficient and cost-effective advanced manufacturing techniques need to be developed and optimized to facilitate the use of bioprinted skin for clinical burn reconstruction. Bioprinted human physiologically relevant skin for burn reconstruction should include different cell types. Active monitoring of cell yields and maintenance of quality parameters such as purity, potency, and viability for the different cell types during production is critical for clinical translation of bioprinted skin [76]. Also, since the bioinks contain ECM scaffold components, the quality of the scaffolds and potential for causing contamination and disease transmission will need to be checked along with real-time monitoring. Non-invasive release testing procedures will need to be established before the delivery of the bioprinted tissues to the patient [99]. Also, to successfully translate organ bioprinting to the clinic, robust automated protocols and procedures need to be established.

To ensure effective use of bioprinted skin for burn reconstruction standards for quality assurance of bioinks, bioprinters and bioprinted products are essential. A comprehensive regulatory framework involving quality control standards for every step of the process—design of the model, selection of bioinks, bioprinting process, validation of the printing, post-printing maturation, and product quality assessment prior to transplantation—is essential. The Food and Drug Administration (FDA) recently issued a guidance document on “Technical Considerations for Additive Manufactured Devices” for production of medical devices [100]. All criteria applicable to engineered tissue will apply to bioprinted skin [91].

Tissue-engineered skin is typically considered as a combination product. Combination products include pharmaceuticals, medical devices, biologics, and their use involves the application of surgical procedures. New surgical procedures are not regulated by the FDA but by the Department of Health and Human Services and can be used on an “as needed” basis at the discretion of the concerned surgeon. However, surgically implantable engineered tissues, depending on their composition, are regulated by the FDA either as devices or biologics and need to be tested in clinical trials before a surgeon is allowed to use them. Currently, products that use stem cells or are derived from stem cells are treated by the FDA as somatic cellular therapies and are regulated as “biologics” under Section 351 of the Public Health Act [91]. As cellular therapies, they are also subject to FDA guidelines for the manufacture of human cells, tissues, and cellular- and tissue-based products found in part 1271 of the same act. Part 1271 establishes the requirements for donor eligibility procedures not found in the current Good Manufacturing Practices (GMP) guidelines of parts 210 and 211 [91]. These guidelines regulate the way stem cells are isolated, handled, and labeled. Also, engineered tissues typically used in research do not require FDA approval during animal and in vitro testing if they are not intended for use on humans. However, Title 21 of the Federal Code of Regulations defines certain restrictions with regard to shipping and disposal of these products.

## Conclusions

Skin bioprinting technology has huge potential to facilitate fabrication of physiologically-relevant tissue and enable better and more consistent functional outcomes in burn patients. The use of bioprinting for skin reconstruction following burns is promising, and bioprinting will enable accurate placement of all the different native skin cell types and precise and reproducible fabrication of constructs to replace injured or wounded skin. The use of 3D bioprinting for wound healing will facilitate faster wound closure, which is critical in the case of extensive burn injuries. Earlier intervention will reduce the potential for infections and contribute to faster healing, reduced scarring, and better cosmetic outcomes. This will also contribute to a reduction in the number of surgeries required and the length of stay in the hospital for patients. To facilitate successful clinical translation and use of bioprinting for wound reconstruction, the developed wound product should be simple and able to seamlessly integrate into the surgical workflow and operative process. Further advances in terms of development of standardized clinical grade 3D bioprinters and biocompatible bioinks will enable wider use of this technology in the clinic. Also, establishment of GMP-compliant cell manufacturing centers allied to medical facilities will facilitate wider adoption of this technology for wound reconstruction. This will also significantly aid in logistics and application of the technology. Overall, 3D bioprinting is a very transformative technology, and its use for wound reconstruction will lead to a paradigm shift in patient outcomes.

## Abbreviations

3D: Three dimensional
AFSC: Amniotic fluid-derived stem cells
CAD: Computer-aided design
CAM: Computer-aided manufacturing
CT: Computed tomography
DLP: Digital light processing
ECM: Extracellular matrix
FDA: Food and Drug Administration
FTSG: Full-thickness skin graft
GMP: Good manufacturing practice
ITOP: Integrated tissue and organ printer
MRI: Magnetic resonance imaging
PCL: Polycaprolactone
STL: STereoLithography
STSG: Split-thickness skin graft
TPP: Two-photon polymerization
